# Intruders or protectors – the multifaceted role of B cells in CNS disorders

**DOI:** 10.3389/fncel.2023.1329823

**Published:** 2024-01-10

**Authors:** James W. Aspden, Matthew A. Murphy, Rommi D. Kashlan, Yueyue Xiong, Mark C. Poznansky, Ruxandra F. Sîrbulescu

**Affiliations:** ^1^Vaccine and Immunotherapy Center, Massachusetts General Hospital and Harvard Medical School, Boston, MA, United States; ^2^Department of Neurology, Massachusetts General Hospital and Harvard Medical School, Boston, MA, United States

**Keywords:** B cells, central nervous system, brain injury, neurodegenerative, inflammation, neuroprotection

## Abstract

B lymphocytes are immune cells studied predominantly in the context of peripheral humoral immune responses against pathogens. Evidence has been accumulating in recent years on the diversity of immunomodulatory functions that B cells undertake, with particular relevance for pathologies of the central nervous system (CNS). This review summarizes current knowledge on B cell populations, localization, infiltration mechanisms, and function in the CNS and associated tissues. Acute and chronic neurodegenerative pathologies are examined in order to explore the complex, and sometimes conflicting, effects that B cells can have in each context, with implications for disease progression and treatment outcomes. Additional factors such as aging modulate the proportions and function of B cell subpopulations over time and are also discussed in the context of neuroinflammatory response and disease susceptibility. A better understanding of the multifactorial role of B cell populations in the CNS may ultimately lead to innovative therapeutic strategies for a variety of neurological conditions.

## 1 Introduction

**FIGURE 1:**
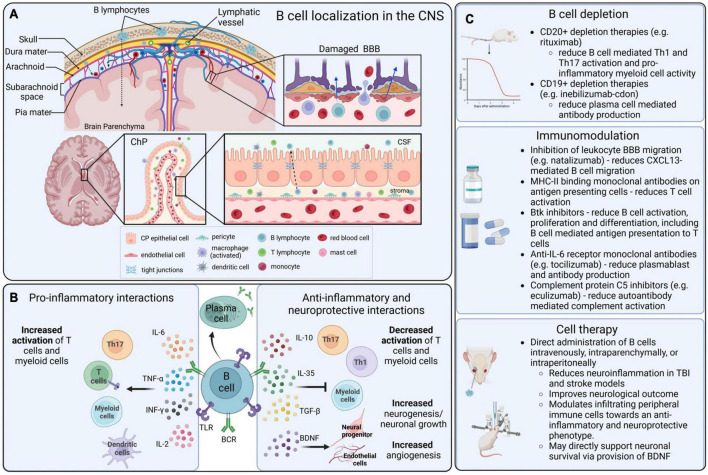
B cell localization, functions, and therapeutic interactions in the CNS. **(A)** B cells that infiltrate into the CNS can originate in the local bone marrow of the calvarium, or from peripheral blood circulation. Lymphocytes can infiltrate into the CNS parenchyma through a damaged blood–brain barrier (BBB), the blood–cerebrospinal fluid barrier (BCSFB) of the choroid plexus (ChP) and circumventricular organs (CVOs), and the blood–meningeal barrier. **(B)** In the CNS, B cells can become activated via surface receptors, including BCRs and TLRs, and produce a variety of pro-inflammatory and anti-inflammatory cytokines that mediate complex immunomodulation in adjacent immune cells, particularly in T cells and myeloid populations. **(C)** Therapeutic strategies that are directly or indirectly mediated by B cells in the context of CNS disorders include B cell depletion strategies, modulatory drugs and biologics that alter the function of B cells, and direct application of B cells as an immunomodulatory cell-based therapeutic.

B cells were initially identified while searching for the source of antibodies, and they are still largely defined by their role in immunoglobulin production (LeBien and Tedder, 2008). The variety of modulatory and regulatory cellular interactions that B cells can engage in has only been explored comparatively recently (Mauri, 2021). In the central nervous system (CNS), long considered an immune privileged site, where infiltration of circulating immune cells is limited, B cells have been associated with a variety of pathologies, most notably immune-driven conditions such as multiple sclerosis (MS) and neuromyelitis optica (NMO), and these conditions have driven our understanding of B cells in injury and pathology (Sabatino et al., 2019; Jain and Yong, 2022). Conversely, some B cell subsets are potent immunomodulators able to minimize neuroinflammation, leading to a reduction in the typical neurodegeneration that follows CNS injury (Rosser Elizabeth and Mauri, 2015; Catalán et al., 2021). Remarkably, B cells can produce bioactive molecules which directly support neuronal survival, such as brain-derived neurotrophic factor (BDNF) and recent studies have identified neuro- and angiogenesis following B cell supplementation (Kerschensteiner et al., 1999; Edling et al., 2004; Sîrbulescu et al., 2017). Collectively, this suggests that B cells could induce or support a neuroprotective environment through immunomodulation and the direct or indirect expression of pro-regenerative factors (Figure 1).

### 1.1 B cell subsets and functions

**FIGURE 2:**
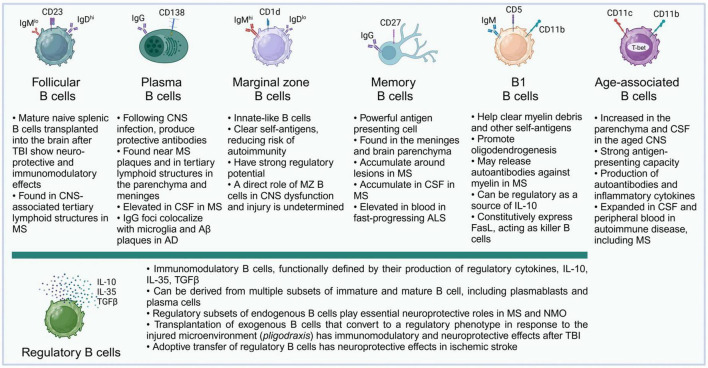
B cell subsets and their described functions in the CNS. Almost all major subsets of mature B cells have been described in the literature in connection with acute or chronic pathologies of the CNS. It is likely that future studies will further add to the growing list of B cell subsets and their complex roles in their interactions with the molecular microenvironment and specific cell subsets in the CNS. For illustration, surface markers typical of murine B cell subsets are shown.

B cells can perform a diversity of cellular and humoral functions depending on their stage of differentiation and activation. Originating from progenitor cells in the fetal liver and the adult bone marrow, B lymphocytes undergo multiple stages of development, and complex maturation in the peripheral lymphoid organs (LeBien and Tedder, 2008; Maddaly et al., 2010). The broad diversity of B cell populations has increasingly been recognized, and while conventional follicular B cells are the predominant recirculating B cell population and the main source of antibody production, a variety of non-canonical B cell subsets have been described (Haas, 2023) (Figure 2). These include B-1 cells, an innate population of B cells found mostly in serosal cavities; marginal zone (MZ) B cells, innate B cells which can respond rapidly, in a T-cell independent manner, to blood-borne and commensal pathogens through the production of IgM and class-switched immunoglobulin G (IgG) and IgA antibodies; regulatory B cells (Bregs), a broad spectrum of B cells characterized by their ability to produce immunosuppressive cytokines; killer B cells, which express high levels of Fas-ligand and can induce apoptosis in T cells and other cell types; and age-associated B cells (ABCs), atypical memory B cell populations, characterized by expression of T-bet and CD11c, and thought to promote inflammatory responses (Cerutti et al., 2013; Haas, 2023).

Antibody production has largely been viewed as the primary function of B cells, both in health and disease. However, B cells can also act as professional antigen presenting cells (APCs), a role which has been studied extensively in the context of cancer (Rossetti et al., 2018; Rastogi et al., 2022). CD40-activated B cells can directly expand antigen-specific CD8+ T cells (Zentz et al., 2007), and mice vaccinated with tumor antigen-pulsed CD40-B cells showed a significant reduction in tumor growth and size (Ritchie et al., 2004). Compared to dendritic cells (DCs), B cells have a greater migratory capacity and can induce persistent PD-1 expression in T cells, which enhances anti-tumor responses (Gonzalez et al., 2015; Zahm et al., 2017). Therefore, B cells may be more effective APCs than DCs in certain contexts, and an interesting therapeutic target. Furthermore, antigen presentation in the CNS by B cells may be responsible for driving neuroinflammation and autoimmune disease. B cell mediated activation of T cells has been documented *in vivo* and is an ongoing topic of research to identify new therapeutics (Molnarfi et al., 2013; Parker Harp et al., 2015).

Independently from antibody production, B cells can be potent immune modulators through the secretion of a range of anti- and pro-inflammatory mediators and cytokines (Vazquez et al., 2015). Numerous studies have highlighted the capacity of B cells to promote inflammatory phenotypes in T cells and myeloid populations through tumor necrosis factor alpha (TNF-α) and interleukin-6 (IL-6) production (Duddy et al., 2004; Arango Duque and Descoteaux, 2014; Vazquez et al., 2015). B cells are also capable of producing IL-10, transforming growth factor beta (TGF-β), and IL-35 – all of which have been demonstrated to exhibit anti-inflammatory effects (Rosser Elizabeth and Mauri, 2015). In the last two decades, scientific understanding of this immunosuppressive function of Bregs has increased substantially (Mauri and Bosma, 2012). Bregs can emerge during almost every stage of B cell development making them challenging to characterize using surface markers. A definition of Bregs is further complicated by the differing locations and expression profiles between species (Rastogi et al., 2022). A widely accepted interpretation of Bregs is the ability to produce at least IL-10 and/or TGF-β, and IL-35, although this is likely to refine further as immunophenotyping improves (Rosser Elizabeth and Mauri, 2015). Local microenvironments provide activating cues to B cells, and the nature and sequence of such cues can influence their cytokine expression profile (Duddy et al., 2007). Activation through B-cell receptors (BCRs) (Matsumoto et al., 2011; Miles et al., 2012) as well as through Toll-like receptors (TLRs) (Lampropoulou et al., 2008; Mauri and Bosma, 2012) has been shown as crucial to IL-10 induction and the regulatory phenotype of B cells. Interestingly, some evidence shows that B cells first activated with TLR agonists amplify IL-10 secretion after restimulation via the BCR pathway, supporting the concept that stepwise staged stimulation may be required for strong IL-10 induction (Matsumoto et al., 2011). Recent studies that applied B cells therapeutically have further demonstrated that expression of modulatory cytokines occurs via myeloid differentiation factor 88 (MyD88)-dependent signaling pathways (Sîrbulescu et al., 2021), a key mediator of TLR signaling. In the context of injury and disease, there are a range of danger-associated molecular pattern molecules (DAMPs) that can interact with TLRs, stimulating cytokine production. Importantly, in injury, B cells can express both anti- and pro-inflammatory cytokines, potentially fluctuating between states and modulating the microenvironment to reduce inflammation (Sîrbulescu et al., 2019, 2021; Dwyer et al., 2023). This concept is particularly relevant when discussing exogenous administration of B cells as a therapeutic strategy.

### 1.2 B cell recruitment into the CNS

Most of our understanding of mechanisms of B cell recruitment in the CNS is derived from studies of pathological states, such as MS, since in the healthy CNS parenchyma B cells are rarely present, and similarly in the cerebrospinal fluid (CSF) of healthy individuals, they represent only a minor fraction of infiltrating immune cells (de Graaf et al., 2011). B cells can extravasate and infiltrate into tissues via classical transmigration, a sequence of rolling, arrest, crawling, and migration (Takeshita and Ransohoff, 2012). Integrins and selectins, including very late antigen-4 (VLA-4) and P-selectin glycoprotein ligand-1 (PSGL-1) bind vascular CAM (VCAM-1) and P-selectin on endothelial cells, which initiates the rolling and slowing down of leukocytes. These weak affinity interactions prepare cells for transmigration across membranes (Takeshita and Ransohoff, 2012). The influence of different cell surface molecules in the context of B cell transmigration has been investigated through knockout studies and antibody blocking, both *in vitro* and *in vivo* (Jain and Yong, 2022). Studies showed that L-selectin had limited importance, while activated leukocyte cell adhesion molecule (ALCAM) deficiency on B cells was associated with reduced recruitment into the CNS (Michel et al., 2019). The transmigration of B cells is initiated by the presence of chemokines and chemo-attractants, such as CC-chemokine and CXC-chemokine ligands (CXCL) (Alter et al., 2003). Chemokines in the microenvironment, including CXCL12, CXCL13, and CXCL10, lead to the upregulation of molecules such as intracellular adhesion molecule 1 (ICAM-1), VLA-4, and lymphocyte function-associated antigen 1 (LFA-1) – all of which are involved in B cell diapedesis in mice (Alter et al., 2003; Lehmann-Horn et al., 2015; Michel et al., 2019; van Langelaar et al., 2019). VLA-4 deletion led to a reduction in B cell CNS recruitment in mouse models of MS, however a similar effect was not observed in patients treated with anti-VLA-4 antibodies (Lehmann-Horn et al., 2015; Häusler et al., 2021). Such discrepancies may point to redundant mechanisms and highlight the importance of investigating molecular pathways across species.

### 1.3 Routes of B cell infiltration into the CNS

Three major routes of access for peripheral immune cells to the CNS include the blood–brain barrier (BBB) and the blood–spinal cord barrier (BSCB), the blood–cerebrospinal fluid barrier (BCSFB) of the choroid plexus (ChP) and circumventricular organs (CVOs), and the blood–meningeal barrier (Mastorakos and McGavern, 2019) (Figure 1).

The meninges are a key point of entry for circulating immune cells as the blood vessels of the dura mater lack tight junctions and are therefore permissive to extravasation (Mastorakos and McGavern, 2019). Within the dural meninges, B cells are constitutively present, representing 15%–30% of all CD45*^hi^* cells (Korin et al., 2017). Recent studies have characterized a lymphopoietic niche, bordering the CNS, that is a local source of meningeal B cells (Brioschi et al., 2021). These cells are able to transverse from the niche to the meninges through ossified vascular channels (Ren and Liu, 2023). While the majority of B cells in the meninges are tissue resident, the presence of peripheral B cells has been shown to increase with age, due to greater accumulation of ABCs (Brioschi et al., 2021). In the mouse healthy CNS, most mature B cells are IgM+ cells with unmutated BCRs but a fraction of IgA+ cells have also been identified (Brioschi et al., 2021; Schafflick et al., 2021). In humans, the phenotype of CNS B cells is not well defined. Some studies have indicated the presence of IgA+ cells in MS which may be gut derived but the lack of in-depth immunophenotyping limits our understanding of the healthy brain (Pröbstel et al., 2020). Before the B cells in the dura can enter the sub-arachnoid space (SAS), they must first transverse the arachnoid mater which is part of the BCSFB. Joined by tight junctions, the arachnoid mater is relatively impermeable to paracellular movement, but it is thought that the structure is altered in disease states, leading to increased cell infiltration (Derk et al., 2021). The SAS sits between the leptomeninges and contains a network of blood vessels surrounded by arachnoid trabeculae. The endothelial cells of these vessels are connected through tight junctions and covered by the pia mater but lack astrocytic feet processes (Mastorakos and McGavern, 2019). As the pia mater does not form tight junctions, peripheral cells can migrate into the CSF, crossing the blood–meningeal barrier. Within the SAS, a recent study has identified a new layer of the meninges – the subarachnoid lymphatic-like membrane (Møllgård et al., 2023). It is postulated that this membrane subdivides the SAS into two compartments which may limit the movement of meningeal cells to the CNS parenchyma. Further research is required to ascertain the role of SAS compartmentalization in disease and injury.

While the CNS is considered an immune privileged site, under inflammatory conditions the BBB may become permeabilized and allow access to lymphocyte infiltration into the parenchyma (Jain and Yong, 2022). The BBB is comprised of blood endothelial cells, basement membrane, and the glia limitans (Mastorakos and McGavern, 2019). The BBB endothelial cells are joined through tight and adherens junctions which collectively limit paracellular transport of solutes and migration of cells (Mastorakos and McGavern, 2019). Diapedesis across the BBB is very limited due to the low physiological expression of cell adhesion molecules ICAM-1 and ALCAM (Cayrol et al., 2008). Neuroinflammation leads to the upregulation of cell adhesion molecules and polarization of resident immune cells which permeabilizes the BBB and increases lymphocytic movement (Jain and Yong, 2022). The endothelium is surrounded by a dense matrix of laminin, proteoglycans, and collagen that contributes to the BBB and limits extravasation. Astrocytes in the parenchyma extend processes that interact with the endothelial basement membrane and form the glia limitans. In a healthy state, tight junctions between these processes offer a further barrier to CNS entry. However, during inflammation, the presence of inflammatory mediators can reduce the expression tight junction components and increase the permeability of this layer (Banks et al., 2012). At the level of arterioles and capillaries, the close association of the endothelial and astrocytic basement membranes prevents the transmigration of immune cells into the CNS. However, at the post-capillary venules, activated leukocytes have been shown to migrate into the perivascular spaces and interact with resident immune cells via major histocompatibility complex (MHC) II interactions (Esiri and Gay, 1990; Mrdjen et al., 2018). This activation leads to enhanced production of matrix metalloproteinases that breakdown the astrocytic endfeet, increasing the permeability of the glia limitans. Moreover, chemoattractants within the inflamed parenchyma may promote egress from the perivascular spaces (Eggers et al., 2017). An alternative point of entry to the CNS parenchyma, relevant for numerous neurodegenerative diseases, is the BSCB which separates peripheral circulation from the CSF and spinal cord parenchyma (Bartanusz et al., 2011). While the structural organization of the BSCB is similar to that of the BBB, the BSCB exhibits a greater permeability to cytokines and potentially to immune cells, attributed to the differential expression of tight junctions and the lower number of pericytes (Ge and Pachter, 2006; Winkler et al., 2012). The presence of peripheral cytokines within the spinal cord parenchyma may therefore act as a catalyst for immune infiltration, both in health and disease.

The third route of access to the CNS from the periphery is via the BCSFB of the ChP. A highly vascularized area, the ChP is responsible for CSF production and an essential component of the neuroimmune system (Strazielle and Ghersi-Egea, 2000; Solár et al., 2020). The capillary network present in the ChP is fenestrated, meaning that peripheral cells can infiltrate and accumulate in the stroma (Strazielle and Ghersi-Egea, 2000). Encompassing the stroma is a sheath of ependymal cells, connected through tight junctions which makes the barrier highly selective to both paracellular diffusion and cellular movement (Thompson et al., 2022). These cells do express ICAM-1 and VCAM-1 which play a significant role in leukocyte adhesion (Steffen et al., 1996; Wolburg et al., 1999). The ligand for L-selectin, has also been characterized in the rat choroidal epithelium (Tamatani et al., 1993). The presence of these molecules highlights the selective nature of the BCSFB as some lymphocyte subsets express receptors enabling transmigration. While the complexity of lymphocyte transmigration across the BCSFB is yet to be fully explained, there is similarity to the process of leukocyte extravasation across the BBB (Takeshita and Ransohoff, 2012). The CVOs border the third and fourth ventricles, assuming essential homeostatic and neurosecretory functions. Similar to the ChP, the blood vessels lack tight junctions and are therefore open to peripheral circulation (Mastorakos and McGavern, 2019). Surrounding these organs is a perimeter of dense astrocytic processes that prevents interaction with surrounding brain regions (Morita et al., 2016). The CVOs are an understudied site of infiltration, and there is limited knowledge on the mechanisms of lymphocyte transmigration. Some studies have shown an upregulation of endothelial ICAM-1 and VCAM-1 in the CVO microvasculature during experimental autoimmune encephalomyelitis (EAE), an animal model of MS (Schulz and Engelhardt, 2005). This was associated with an increased number of CD45+ leukocytes in the CVOs. CVO involvement as a site of neuroinflammation in EAE has also been demonstrated using *in vivo* magnetic resonance imaging (MRI) using a gadolinium-based contrast agent, further confirming a role in the neuroimmune system (Wuerfel et al., 2010). The cell adhesion molecules involved are comparable to those seen in leukocyte transmigration and it is likely that B cells utilize a similar mechanism to enter the CVOs, although more research is required to confirm this hypothesis.

## 2 B cells in chronic and neurodegenerative pathologies of the CNS

In recent years, B cell involvement in chronic neurodegenerative disease, whether inflammatory or regulatory, has been a topic of great interest and some controversy. Conditions such as MS (Comi et al., 2021) and NMO (Bennett et al., 2022) are prime examples, where B cells are thought to drive disease development and progression. However, the mechanisms involved remain only partially understood. Here we aim to collate knowledge around this area, describing the presence of B cells in disease, their entry into the CNS, how they influence pathophysiology and B cell targeting therapeutics. We include diseases that are not typically associated with B cells, such as Alzheimer’s disease (AD), Parkinson’s disease (PD), and amyotrophic lateral sclerosis (ALS), as inflammation has increasingly been shown to play a role in the progression of these conditions.

### 2.1 Multiple sclerosis

Multiple sclerosis is a debilitating chronic autoimmune condition, characterized by neuronal injury, demyelination and inflammation, resulting in functional loss and reduced quality of life (Calahorra et al., 2022). B cells involved in MS pathogenicity mature in the periphery, via antigen presentation within cervical lymph nodes (CLNs). CLNs drain the CNS, exposing B cells to lesion antigens. This also creates a route for B cell migration into the CNS and MS lesions. High throughput sequencing on MS autopsy tissue has identified clonally expanded B cells in both CLNs and the CNS. A higher prevalence of clone precursors was present in CLNs, compared to the CNS, while mature clones were found in the CLN, CNS and within lesions (Stern et al., 2014). Though maturation predominately occurs peripherally, further maturation may also take place in the CNS, within tertiary lymphoid organs (TLOs). TLOs are aggregates of immune cells that resemble germinal centers within secondary lymphoid organs, which may facilitate lymphocyte activation and B cell maturation, including antigen presentation, somatic hypermutation, and class switch recombination within B cells (Serafini et al., 2004; Mitsdoerffer and Peters, 2016). TLOs have also been identified in multiple mouse models of autoimmune conditions (Lochner et al., 2011).

#### 2.1.1 B cell migration and localization in MS

In MS, CD20+ B cells have been found primarily in the leptomeningeal SAS, rather than the brain parenchyma (Machado-Santos et al., 2018). Furthermore, in patients with progressive MS, B cells have been found to colocalize with activated microglia in the meninges, especially in subpial cortical regions, correlating with increased disease severity (Magliozzi et al., 2007; Howell et al., 2011; Choi et al., 2012). In the CSF of patients with active MS, the levels of CXCL13, a B cell chemoattractant, are highly increased (Eggers et al., 2017), correlating with the higher aggregation of memory B cells and plasma cells in the CSF. CXCL13 is produced by stromal cells (Kowarik et al., 2012) and is likely to play a pivotal role in attracting B cells, binding the pan-B cell receptor CXCR5 (Bagaeva et al., 2006; Henry and Kendall, 2010). Interestingly, CXCL13 was reduced after treatment with methylprednisolone, used for controlling inflammation in MS relapses, and natalizumab, an inhibitor of leukocyte migration into the brain (Hutchinson, 2007). This indicates a potential link between reduced CXCL13 levels, decreased B cell migration into the CNS and reduced disease activity (Sellebjerg et al., 2009). B cell recruitment may also be influenced by other immune cells, releasing chemoattractants and other cytokines, such as self-reactive CD4+ T cells that have evaded negative selection and are thought to migrate across a disrupted BBB, facilitating B cell recruitment (Dendrou et al., 2015).

Another possible route of access is via the CVOs, which do not possess a BBB (Miyata, 2022). In EAE, increased lesions were visualized by Gadofluorine M- (Gf) enhanced MRI scans in the ChP and surrounding areas which correlated with disease severity. Furthermore, histological examination showed that Gf was internalized by macrophages/microglia, demonstrating that they were able to access the CVOs (Wuerfel et al., 2010). However, whether B cells can gain access via this route is unclear. Increased numbers of B cells have been detected in the brain and spinal cord in EAE models, but not in the ChP (Lazarevic et al., 2023).

#### 2.1.2 B cell antibody-dependent impact in MS

Immunoglobulin G oligoclonal bands (OCBs) are found extensively in the CSF of patients with MS, and have been detected intrathecally within lesions (Gilden et al., 1978). Despite this, the role of antibodies in disease pathology remains unclear and is somewhat controversial (Yu et al., 2020). Antibodies were thought to be a hallmark of MS and though they have been used diagnostically for some time, they are not pathognomonic in MS (Deisenhammer et al., 2019; Ganelin-Cohen et al., 2023). They are produced by mature CD19+ B cells, including plasma cells, which have been detected in the parenchyma (Pollok et al., 2017) and perivascular space of patients with chronic MS (Prineas and Wright, 1978). These cells are CD20- and are therefore not targeted with CD20-depletion therapies, such as Rituximab. The success of these therapies in reducing disease burden indicates that antibodies are not the sole drivers of disease progression. However, the marginal efficacy of CD20-depletion therapies in progressive MS suggests autoantibodies may be implicated in this subtype. This may be through enhancing complement-mediated cytotoxicity, which was found to be increased within post-mortem brain tissue within cortical gray matter lesions (Watkins et al., 2016).

Patients with densely populated IgG lesions showed a better response to therapeutic plasma exchange during an attack compared to other patterns of disease, indicating a potential role of autoantibodies in pathogenesis (Keegan et al., 2005). *In vitro* bioassay studies have examined the effect of serum-derived patient IgG in a myelinating culture system and found complement-dependent demyelination by IgG in around 30% of patients with MS (Sorensen et al., 2008). Furthermore, immunofluorescence microscopy revealed IgG bound specifically to myelinating oligodendrocytes (Elliott et al., 2012). This indicates that antibody-dependent mechanisms may influence disease pathology (Derfuss et al., 2010; Roshan et al., 2023). The lack of consistency between tissue culture and imaging methods makes drawing definitive conclusions about the role of autoantibodies difficult (Yu et al., 2020).

In contrast, some studies have suggested autoantibodies may be involved in CNS remyelination (Rodriguez and Lennon, 1990; Asakura and Rodriguez, 1998; Warrington et al., 2000). Other inferences have been drawn due to incomplete effect of anti-CD20 therapies (Lehmann-Horn et al., 2017) and the effect of laquinimod, a T cell therapy which influences B cell responses and inhibits MOG-specific IgG antibodies (Varrin-Doyer et al., 2016). Furthermore, numerous existing studies make the association to the presence of antibodies, rather than proving direct effects in pathophysiology (Link and Huang, 2006). OCBs have also been shown to react to ubiquitous self-proteins and intracellular antigens, indicating their activity may be in response to injury, instead of a primary cause (Brandle et al., 2016). Whether autoantibodies initiate MS or are a response to cellular damage remains unknown and therefore further evidence is required to better understand pathophysiology and guide future treatments.

#### 2.1.3 B cells as antigen-presenting cells in MS

B cells play a critical role as APCs in MS pathogenesis, demonstrated primarily by their interactions with T cells. Mice with B cells that lacked MHC-II, and thus could not present antigen to T cells, did not develop EAE when stimulated with recombinant human myelin oligodendrocyte glycoprotein (rhMOG). In these mouse models, B cell antibody secretion was also inhibited, indicating that the interaction between B cells and T cells is crucial for disease development. Th1 and Th17 T cell responses, which mediate neuroinflammation, BBB disruption and activation of other proinflammatory cells, were also diminished (Prajeeth et al., 2017; Balasa et al., 2020). Moreover, Th17 reduction is seen in the context of CD20 depletion therapies (Bar-Or et al., 2010), further connecting the role of B cells in activating pathogenic T cell responses.

In a B cell dependent, antibody independent EAE model, stimulated by a peptide encompassing the extracellular domains of myelin proteolipid protein (PLPECD), it was shown that depletion of B cells caused significant inhibition of this peptide’s ability to generate EAE (Wilhelm et al., 2023). CD4+ T cell activation, proliferation and proinflammatory cytokine production were also inhibited, and when CD4+ T cells extracted from B cell deficient mice were stimulated with PLPECD, they were unable to replicate EAE in naïve models. B cells defective in MHC-II again failed to replicate the disease, further evidencing the role of B cells as APCs in EAE. Interestingly, in separate cultures, WT B cells stimulated even greater antigen-mediated PLPECD-reactive CD4 T cell proliferation than DCs (Wilhelm et al., 2023), which are professional APCs (Sung, 2019). The importance of B cells as APCs has also been indicated in patients, with myelin reactive B cells expressing greater levels of costimulatory molecules such as CD80 and CD86 (Aung and Balashov, 2015), necessary for CD4+ T cell activation and a proinflammatory phenotype associated with increased interferon (IFN) production, compared with healthy controls (Harp et al., 2010; Kinzel and Weber, 2016). Indeed, teriflunomide treatment in patients with relapsing-remitting MS (RRMS) resulted in downregulation of B cell CD80 and CD86 expression, reducing pathogenicity (Wu et al., 2023).

#### 2.1.4 B cells as proinflammatory mediators in MS

In patients with MS, there is an immunoregulatory imbalance resulting in increased proinflammatory and reduced anti-inflammatory cytokine production in B cells. B cells can produce a variety of classical inflammatory cytokines including IL-6, TNF-α, GM-CSF, and lymphotoxin A (LTa), that can drive MS pathology (Li et al., 2015a). IL-6 deletion can prevent the development of neurological symptoms in a mouse model of EAE, indicating a key role for this cytokine, potentially via a reduction in Th1 and Th17 T-cell differentiation (Serada et al., 2008; Molnarfi et al., 2013). Interestingly a subset of activated B cells, expressing CD137, have recently been described to concentrate in meningeal infiltrates within brain samples from patients with MS (Wong et al., 2020). Upon stimulation with the ligand CD137L, CD137+ B cells demonstrate significantly increased IL-6 production (Wong et al., 2020). CD137L is expressed by professional APCs which could stimulate CD137+ B cells, further amplifying this proinflammatory response.

Both naïve and memory B cells from MS patients have a disproportionate expression of IL-6 and reduced IL-10 response, as compared to healthy controls (Ireland et al., 2012). IL-6 can drive a Th17 phenotype from naïve T cells (Bettelli et al., 2006), and reduce regulatory T cells (Tregs) (Korn et al., 2008; Kimura and Kishimoto, 2010; Schneider et al., 2013) in EAE and MS patients. Evidence also suggests a connection with B cell IL-6 production as IL-6 deficient B cells significantly reduce Th17 production in EAE mice compared with B cell WT controls (Barr et al., 2012). Conversely, enhanced signaling through the IL-6 receptor increases B cell antigen presentation to T cells, increasing T cell cytolytic inhibition and suppression of IL-10 mediated effects and driving pathogenic Th17 differentiation (Ireland et al., 2015). It is also important to consider IL-6 can have pleiotropic roles. It has also been shown to support Th2 differentiation in T cells by inducing expression of IL-4 (Rincón et al., 1997), and to reduce Th1 differentiation by blocking IFN-γ signaling (Diehl et al., 2000).

In B cells isolated from the peripheral blood of patients with RRMS, stimulation with self-antigen myelin basic protein (MBP) increased expression of both TNF-α and IL-6 (Nielsen et al., 2016). TNF-α expression was positively correlated with disease severity in patients, according to the expanded disability status scale, demonstrating an increased proinflammatory profile of B cells in MS patients. Furthermore, B cells from patients with RRMS showed reduced expression of IL-10, which correlated with more aggressive disease. This indicates a diminished immunoregulatory function of B cells in these patient groups (Nielsen et al., 2016).

MicroRNAs are short single-strand oligonucleotides that post-transcriptionally modulate gene expression and show cellular importance in modulating cytokine production within B cells (Miyazaki et al., 2014). Overexpression of miR-132 correlates with increased TNF-α and LTa expression in MS patients, via suppression of sirtuin-1. Overexpression of miR-132 in normal B cells replicated this finding, upregulating TNF-α and LTa levels, which could be inhibited by resveratrol treatment, a sirtuin-1 activator, indicating a potential mechanism for aberrant inflammatory cytokine production in MS (Miyazaki et al., 2014). However, there have also been reports of resveratrol exacerbating disease in EAE models (Sato et al., 2013) with other studies concluding the proposed activation of sirtuin-1 by resveratrol is an artifact (Kaeberlein et al., 2005).

#### 2.1.5 Neuroprotective roles of B cells in MS

B cells have complex regulatory functions, producing cytokines that modulate the amplitude of inflammatory responses, including IL-10, IL-35, and TGF-β (Shen and Fillatreau, 2015). IL-10 activity is mediated through the JAK1 and Tyk2 pathways, stimulating phosphorylation and subsequent activation of STAT1, STAT3, STAT5, and SOCS3 resulting in nuclear factor kappa-B (NF-κB)-orchestrated signal transduction inhibition (Moore et al., 2001). This culminates in modulation of pro-apoptotic genes and inhibition of cell activation, cytokine production and proliferation (Hu et al., 2007).

Bone marrow transplantation studies replacing WT B cells with IL-10 deficient B cells exacerbates disease in EAE mice (Fillatreau et al., 2002; Maheshwari et al., 2023). Chimeric mice lacking IL-10 in B cells had non-remitting severe disease, with >50% mortality at day 30 (Skok et al., 1999), and this severe phenotype could be rescued through adoptive transfer of IL-10-competent splenic B cells from B6 mice (Fillatreau et al., 2002; Lampropoulou et al., 2008).

In patients with MS, Duddy et al. have previously shown that B cell expression of IL-10 is diminished. B cells isolated from peripheral blood were stimulated *in vitro* by BCR and CD40 activation, mimicking both an antigen response and T cell activation. There was markedly less IL-10 production in MS B cells compared to age and sex matched controls (Duddy et al., 2007). Naïve B cells are responsible for the majority of IL-10 production in healthy B cells populations, with memory B cell being largely implicated in inflammatory cytokine production (Duddy et al., 2007). This is especially relevant in the context of depletion therapies as those that selectively target memory B cells, or increase naïve B cell production could be critical in controlling MS disease progression.

#### 2.1.6 Therapies targeting B cells in MS

B cell depletion therapies (BCDTs) which include antibody-mediated depletion or inhibition of peripheral immune cell migration (Brinkmann et al., 2010) have highlighted both the role of B cells in MS progression and their complex immunoregulatory functions (Lee et al., 2021). Rituximab is a key BCDT. A monoclonal antibody targeting CD20+ B cells, it leads to cell-mediated apoptosis (Chisari et al., 2022). It can deplete pre-B and mature B cells, including memory (CD27+) and naïve (CD27−) B cells (Li et al., 2015b; Baker et al., 2017). Interestingly, plasma cells do not express CD20 and even though the presence of OCBs in CSF has been used diagnostically, these are not pathognomonic in MS and their role in disease progression is a topic of debate (Ganelin-Cohen et al., 2023).

Rituximab reduces B cell mediated Th1 and Th17 activation as well as pro-inflammatory myeloid cell activity (Bar-Or et al., 2010; Li et al., 2015a) which is associated with reduced MRI detected disease activity and relapse rate vs. placebo (Hauser et al., 2008). Upon reconstitution post treatment, B cell populations are predominantly naïve (CD27−), exhibiting increased IL-10 production and reduced proinflammatory cytokine production (Barr et al., 2012; Li et al., 2015b), indicating the pathogenic role of memory B cells in MS. This was further demonstrated by Atacicept clinical trials, a drug that acts by blocking B cell development and survival factors including B-cell activating factor (BAFF) and a proliferation-inducing ligand (APRIL) (Mackay and Browning, 2002). This induced a shift toward an increased memory (Tak et al., 2008) and reduced IL-10 B cell phenotype (Yang et al., 2010), which resulted in increased patient relapses and subsequent termination of the trial (Kappos et al., 2014). Additional BCDTs based on selective anti-CD20 monoclonal antibodies, including ocrelizumab (Lin et al., 2022; Margoni et al., 2022) and ofatumumab (Hauser et al., 2022; Kang and Blair, 2022) have gained recent regulatory approval and are well-tolerated clinically. Important considerations on these newer therapeutics reside around inflammatory side effects potentially caused by complement activation (Hauser et al., 2022).

Interestingly, CD20+ depletion therapies have also been shown to target a subset of T cells, found to be elevated in MS, they can acquire CD20+ through trogocytosis, the active transfer of cell surface molecules from one cell to another (Miyake and Karasuyama, 2021). This process is reliant on contact-dependent interaction and antigen presentation between B cells and T cells (Sumida and O’Connor, 2022). In both patients with MS and in EAE animal models CD20+ T cells have been shown to express proinflammatory cytokines, activation markers and adhesions molecules, suggesting pathogenicity (Ochs et al., 2022). Furthermore, adoptive transfer of CD20+ T cells can exacerbate disease severity, while targeted anti-CD20+ T cell depletion can reduce EAE-associated demyelination independent of the presence of B cells (Ochs et al., 2022). These findings underlie the complexity of off target effects in cellular depletion therapies, that may involve additional cellular categories.

Side effects of BCDTs include susceptibility to infection via immunosuppression (D’Abramo et al., 2022; Langer-Gould et al., 2023) and the off-target depletion of Breg populations. These therapies exhibit a lack of specificity for inflammatory B cells, thereby also depleting IL-10 immunoregulatory B cells, which may enhance disease progression.

More nuanced therapies are therefore being developed, with mechanisms of action targeting cellular activation and interaction. Alternative therapeutic approaches to MS include immunomodulatory strategies that entirely avoid B cell depletion. A key example is glatiramer acetate (GA), an analog of MBP (Ireland et al., 2015) that acts by binding to MHC-II on APCs and disrupting antigen presentation to T cells. Interestingly, a consequence of GA administration is increased IL-10 production in DCs, regulatory monocytes, as well as B cells, enhancing their regulatory function (Begum-Haque et al., 2011) and suppressing EAE progression (Aharoni et al., 2020; Hausler et al., 2020). Studies in patients with MS receiving GA treatment confirmed a direct impact of GA on B cells, with reduced levels of activation markers and proinflammatory cytokines and increased IL-10 secretion and MHC-II expression (Hausler et al., 2020).

Alternative depletion strategies include other cellular targets, such as CD52, a cell surface antigen expressed at high levels on the surface of T and B lymphocytes targeted by alemtuzumab (Katsavos and Coles, 2018; Li et al., 2018) Lymphocyte depletion has also been performed using cladribine, a deoxyadenosine analog prodrug that preferentially depletes lymphocytes, with observed clinical benefits in patients with MS (Deeks, 2018; Rammohan et al., 2020). Another approach involves Burton’s tyrosine kinase (Btk) inhibition. Btk phosphorylation is essential for activation, proliferation and differentiation of B cells after antigen presentation via BCR signaling and therefore regulates T cell antigen presentation (Carnero Contentti and Correale, 2020). Btk inhibitors reduce, rather than switch off Btk function, which could be useful in reducing autoantigen stimulated proinflammatory responses, without leaving patients entirely immunocompromised. In a phase II clinical trial, the Btk inhibitor evobrutinib was shown to reduce slowly expanding lesion volume in patients with MS (Schneider and Oh, 2022). Greater doses caused greater volume reduction over 48 weeks. Future strategies for the treatment of MS will likely involve therapies with improved specificity for proinflammatory pathways, rather than large scale depletion of complex immune cell populations. New therapies, including Btk inhibitors may also play a role as adjuvant therapies, alongside CD20 depletion. This may have a cumulative effect, reducing proinflammatory T cell activation alongside a reduction in memory B cells and an increase in the naïve B cell pool (Lehmann-Horn et al., 2013). Interestingly, anti-CD20 treatment has been shown to be harmful in inactivated (largely naïve) B cell populations, in contrast to the benefit seen in activated populations (Weber et al., 2010). CD20 depletion may also influence the inflammatory profile of other immune cells, with a reduction of B cell IL-10 regulation likely responsible for proinflammatory monocyte differentiation in EAE mice receiving anti-CD20 treatment (Lehmann-Horn et al., 2011). More studies are also warranted in patient cohorts, to see if Bregs play as large a role in humans with MS as seen in EAE models. If so, increasing their production or reducing loss would also target an unmet clinical need (Lehmann-Horn et al., 2013, 2017).

### 2.2 Neuromyelitis optica

Neuromyelitis optica is an inflammatory autoimmune disorder that targets the CNS, resulting in optic neuritis and transverse myelitis (Carnero Contentti and Correale, 2021). B cells play a pathological role in NMO development and progression through a variety of mechanisms including aquaporin 4 autoantibody (AQP4-ab) production (Saadoun et al., 2010), increased proinflammatory activation, disruption of checkpoints for B cell tolerance, reduced regulatory capacity, and loss of anergy (Bennett et al., 2015). Clinically, a distinguishing feature of NMO compared to MS is the presence of autoantibodies against AQP4, found in >75% of patients (Kawachi and Lassmann, 2017; Janssen et al., 2020). AQP4 is a water channel predominately present on astrocyte foot processes at the BBB (Kawachi and Lassmann, 2017). Antibody binding leads to complement-mediated astrocyte depletion (Jarius et al., 2008; Bennett et al., 2009; Zhang and Verkman, 2013) and consequent BBB permeabilization allowing infiltration of lymphocytes, plasma cells and granulocytes, further driving pathogenesis. Activated CD4+ T cells expressing Ox40 also induce inflammation in the brain of NMO-IgG EAE mouse models, by upregulating proinflammatory cytokines after interaction with professional APCs, contributing to BBB disruption and subsequent autoantibody entry into the brain and CSF (Pohl et al., 2013).

In patients with NMO relapses, the numbers of IgG-producing CD138+/HLA-DR+ plasmablasts were elevated in peripheral blood and enriched in the CSF (Chihara et al., 2013). By sorting plasmablasts from both sources and sequencing the IgG heavy chain’s complementarity-determining regions (CDRs), plasmablast clones from peripheral blood and CSF were determined to have identical CDR sequences, indicating preferential migration of IgG-producing plasmablasts into the CSF (Chihara et al., 2013). After autoantibody infiltration, oligodendrocyte injury, axonal demyelination and neuropathy ensue (Iida and Isobe, 2023). As in MS, analysis of plasmablasts isolated from the CSF of patients with NMO suggests that these cells may undergo local maturation to produce immunoglobulins against AQP4 in the CNS (Kowarik et al., 2017). Intrathecal AQP4-ab production was also detected in patients by live cell staining immunofluorescence assay using a CSF:serum AQP4-IgG ratio and a specific AQP4-IgG index as previously described (Jarius et al., 2010; Dujmovic et al., 2011). It is possible that intrathecal IgG production might reflect a secondary B cell activation, perhaps directed at antigens revealed due to primary tissue damage caused by inflammation or as a result of coexisting conditions. It is important to note that TLOs or follicle-like structures, as seen in MS, have not been detected in the meninges of patients with NMO and therefore sites of local autoantibody production are not well defined. In addition, studies indicate that AQP4-ab production is largely restricted to the periphery (Takahashi et al., 2007; Jarius et al., 2010, 2011; Dujmovic et al., 2011).

Increased number of plasmablasts are found in the blood of patients with NMO, accompanied by raised levels of IL-6 – a cytokine involved in plasma cell differentiation and survival (Icoz et al., 2010). Cytological and protein biochemical analysis of 211 lumbar punctures from 89 patients during acute relapses showed that intrathecal IgG production was minimal, and that CSF infiltration of immune cells included neutrophils, eosinophils, activated lymphocytes and/or plasma cells, which were detected in nearly 50% of patients. This pleocytosis alongside blood-CSF barrier breakdown was maintained in some patients during remission, indicating low level inflammation may occur between relapses (Jarius et al., 2011). Studies have implicated many cytokines in plasma cell survival *in vitro*, including IL-5, IL-6, TNF-α, BAFF, and APRIL (Cassese et al., 2003). The variability in blood and CSF levels of plasma cells and AQP4-ab in patients during acute relapses (Jarius et al., 2008, 2011) indicates that these signals may be involved in regulating AQP4-IgG-ab production at different courses of the disease. Indeed, eosinophils are primary producers of IL-6 and APRIL in the bone marrow and are crucial to the maturation of plasma cell populations (Chu et al., 2011). Similarly, eosinophil presence in the CNS (Correale and Fiol, 2004) and in CNS lesions (Lucchinetti et al., 2002) may enhance plasma cell survival and IgG production in NMO lesions. Controlling eosinophil numbers, location or targeting IL-6 signaling could therefore impact plasma cell survival and AQP4-IgG-ab production, modulating NMO pathology. For instance, the S1P1 receptor agonist fingolimod retains eosinophils in the bone marrow and antibody secreting cells (ASCs) in secondary lymphoid tissues (Kabashima et al., 2006; Sugita et al., 2010). This effect could lead to increased AQP4-IgG levels in the blood and migration of CNS ASCs, explaining the worsening NMO disease activity seen with fingolimod treatment (Min et al., 2012). Natalizumab, an MS treatment known to exacerbate NMO disease activity (Barnett et al., 2012; Kleiter et al., 2012), elevates circulating eosinophils (Polman et al., 2006; Abbas et al., 2011), possibly contributing to lesion formation (Zhang and Verkman, 2013) or supporting local ASCs. Conversely, inhibiting IL-6 signaling with the anti-IL-6 receptor monoclonal antibody tocilizumab, reduces peripheral blood plasmablasts and AQP4-IgG levels in some patients and appears to decrease relapse rates in NMO patients (Araki et al., 2014). These findings suggest IL-6 has a crucial role in inducing plasma cell production of AQP4-IgG-ab (Cassese et al., 2003).

#### 2.2.1 Proinflammatory B cells in NMO

Interestingly, modulation of disease activity can occur without significant AQP4-IgG reduction (Pellkofer et al., 2011), demonstrating a potential role of humoral-independent pathogenesis in NMO. Antigen presentation and cytokine secretion by B cells may mediate T cell responses via MHC-II pathways, as in MS. In turn, this could generate T follicular helper cells, responsible for B cell differentiation and immunoglobulin isotype switching (Mitsdoerffer et al., 2010; Molnarfi et al., 2013). These interactions may be crucial for AQP4-IgG production. Proinflammatory cytokines such as IL-6 produced by B cells may also direct T cell mediated pathogenicity, through Th17 differentiation (Mitsdoerffer et al., 2013). Indeed, increased IL-17A and IL-23 levels have been found within NMO lesions during relapses (Wang et al., 2011). This may in part explain the discrepancy of reduced lesion activity with elevated AQP4-IgG-ab titers, seen in anti-CD20 rituximab treatment (Cree et al., 2005; Kimbrough et al., 2012). Rituximab does not target plasma cells, which lack CD20, indicating possible humoral independent mechanisms related to reduced IL-6 producing B cells in patients with NMO. The reduced memory B cell profile seen after rituximab treatment in MS may also apply to NMO, with reconstituting B cells secreting reduced IL-6 as well as other inflammatory cytokines, including TNF-α and LTa (Bar-Or et al., 2010; Barr et al., 2012). Further studies are needed to definitively determine the role, number, and characteristics of proinflammatory B cells in NMO and related disorders. Raised levels of BAFF, APRIL, CXCL13, and IL-6 have also been detected in the CNS of patients with NMO, which are likely involved in recruiting and stimulating AQP4-ab producing cells (Vaknin-Dembinsky et al., 2010; Kaneko et al., 2018), facilitating pathogenicity (Bennett et al., 2015; Graf et al., 2021).

#### 2.2.2 Neuroprotective roles of B cells in NMO

Regulatory B cells, responsible for mediating immunoregulation, may also be involved in NMO. In EAE mouse models, IL-35 was essential for generating Bregs (Shen et al., 2014; Wang et al., 2014) and mediating inflammatory macrophage and T cell responses (Collison et al., 2007). Mice with IL-35-deficient B cells also failed to recover from EAE. Interestingly, in EAE, plasmablasts and plasma cells were determined to be a main source of B cell IL-10 and IL-35 production (Shen et al., 2014). It is possible that increased plasma cell formation in NMO could be associated with increased regulatory cytokines. The exact status of Breg production in NMO is not known and information related to their immunoregulatory effects is mainly inferred from studies of other conditions, such as MS (Iwata et al., 2011; Miyagaki et al., 2015; Rosser Elizabeth and Mauri, 2015; Figueiro et al., 2016). Therefore, more research is needed into the IL-10 secreting capacity of NMO plasmablasts, including how production changes in AQP4-IgG-antibody producing cells alongside other B lymphocyte populations in disease. Decreased B10 regulatory cells (CD19^+^CD24*^high^*CD38*^high^*) have been observed in relapsing AQP4-IgG-ab producing patients with NMO (Quan et al., 2013). *In vitro* B cell stimulation in these patients also exhibited reduced IL-10 production (Quan et al., 2013). B10 regulatory production could have been underestimated however, as not all regulatory cells express the same markers and reliance on CD38*^high^* expression may neglect other regulatory phenotypes (Iwata et al., 2011). The neuroprotective role of B cells, through production of neurotrophic factors, in NMO requires further exploration.

Interleukin-10 producing B cells, defined phenotypically as CD19^+^CD39^+^CD1d^+^IL-10^+^, have been detected in the peripheral blood of NMO patients via flow cytometry. B10 cell frequency out of total lymphocytes was greater in all patients with NMO compared to healthy controls, when stimulated with PMA and ionomycin. Furthermore, B10 cells were positively correlated with AQP4-ab intensity. This indicates that these immunoregulatory subsets may mediate disease activity in NMO (Cho et al., 2018), similarly to MS (Michel et al., 2014; Habib et al., 2015). Other studies have indicated a reduction in Bregs (Quan et al., 2013), indicating further research is required to provide clarity on the presence and function of Bregs in NMO. Decreased CD19^+^CD24*^high^*CD38*^high^* Bregs and IL-10+ B cells have also been detected in patients with NMO. This variance in IL-10 B cell levels in patients with NMO could be due to different categorization of Bregs or heterogeneity in stimulants used, as well as they type of immunosuppressive treatment administered to study participants. It is hypothesized that increased levels of BAFF and CXCL13 in the CSF of patients with NMO could lead to greater intrathecal recruitment and activation of B cells, however this warrants further investigation to confirm these associations (Quan et al., 2013).

Dysregulated central and peripheral B cell tolerance checkpoints may drive NMO pathogenesis, as seen in other autoimmune conditions. It has been suggested that inappropriate tolerance may drive AQP4-IgG production alongside other self-targeting B cells (Pittock et al., 2008). Further studies exploring this are lacking, however some findings suggest that AQP4-specific T cells may evade central tolerance checkpoints, contributing to NMO pathogenicity (Sagan et al., 2016). Comparison between seropositive AQP4-ab+ patients and AQP4-ab− patients may reveal insights into whether aberrant tolerance checkpoints are responsible for AQP4 targeted autoreactivity. Furthermore, if only peripheral checkpoints are dysregulated, as suspected in MS, this may guide treatment regimens. After anti-CD20 therapy, reconstitution primarily generates naïve B cells, which are more regulatory compared to memory B cells. Therefore, intermittent treatment could be beneficial, generating a greater naïve B cell pool, which could translate to whether continuous or intermitted B cell depletion is required in NMO.

#### 2.2.3 Therapies targeting B cells in NMO

During acute NMO relapses the mainstay of treatment is steroids and plasma exchange (Carnero Contentti and Correale, 2021), as with MS. The well-established involvement of B cells in NMO pathogenesis, via IL-6 and AQP4-ab production, highlights the rationale for anti-CD20 and anti-CD19 therapies, whose efficacy has been demonstrated in various clinical trials (Cree et al., 2019; Tahara et al., 2020). However, despite similarities, many therapies effective in MS are not recommended for use in NMO, including fingolimod and natalizumab, as they may exacerbate the condition (Jacob et al., 2012; Izaki et al., 2013). Alternatively, reducing relapses has predominantly been mediated by steroid treatment and immunosuppressants including azathioprine, mycophenolate mofetil, methotrexate, tacrolimus, and cyclosporine A (Kageyama et al., 2013; Ramanathan et al., 2014; Chen et al., 2017; Wang et al., 2021a,c). The efficacy of the B cell depleting anti-CD20 drug rituximab has also been demonstrated in reducing relapse frequency and neurological deficits in patients with NMO (Tahara et al., 2020; Wang et al., 2021b). More recently, other molecules are gaining interest as potential therapeutic targets in NMO. Monoclonal antibodies directed at complement C5, IL-6R, and B cell CD19 have shown effectiveness in randomized controlled trials (Traboulsee et al., 2020; Romeo, 2021; Nie and Blair, 2022; Fung and Shirley, 2023).

Eculizumab is an FDA approved NMO treatment (Brod, 2020) which inhibits the terminal complement protein C5, preventing cleavage into the proinflammatory C5a and C5b which induce the membrane attack complex (Nishimura et al., 2023). Mouse studies indicate AQP4-ab can induce the complement cascade *in vitro* and *in vivo* (Hinson et al., 2012; Wang et al., 2017) causing astrocyte inflammation due to membrane attack complex activity. This effect was abrogated in mice treated with a complement inhibitor (Hinson et al., 2012). Satralizumab is also an FDA approved treatment for NMO (Heo, 2020), targeting IL-6 receptors (Romeo, 2021) and mediating the pleiotropic effects of IL-6. This includes proinflammatory Th17 cell differentiation (Lin et al., 2016), B cell differentiation into AQP4-ab producing plasmablasts (Chihara et al., 2011), complement regulation (Erta et al., 2012) and increased BBB permeability (Takeshita et al., 2021). IL-6 is elevated in the serum of patients with NMO (Uzawa et al., 2010; Barros et al., 2016) and is positively correlated with disease severity (Matsushita et al., 2013) and AQP4-IgG levels (Uzawa et al., 2010). Inebilizumab-cdon has recently gained FDA approval for treatment of NMO (Ali et al., 2022). It targets CD19, largely expressed in B lineage cells which differs from rituximab in that it can deplete plasma cells, which express CD19, but not CD20. This reduces AQP4-ab production (Bennett et al., 2015). Afucosylation of Inebilizumab-cdon results in almost 10-fold greater affinity for the FcγRIIIA receptor (Yan et al., 2022), key in regulating antibody-dependent cellular cytotoxicity and antibody-dependent phagocytosis (Tullman et al., 2021; Ali et al., 2022). The effectiveness of these therapies in reducing AQP4-ab mediated astrocyte destruction, either through inhibiting plasma cell proliferation or directly altering the effects of AQP4-abs demonstrates the prominent role of B cells in NMO pathogenesis.

### 2.3 Parkinson’s disease

Parkinson’s disease (PD) is characterized by death of dopaminergic neurons in the substantia nigra, resulting in dysregulation of movements including bradykinesia, tremor and rigidity (Furgiuele et al., 2023). Lewy bodies accumulate composed of inappropriately folded α-synuclein, which aggregate within neurons, further contributing to neuronal death and pathogenesis. Greater levels of IL-6, TNF-α, IL-1b, IL-2, IL-10, C-reactive protein, and RANTES were also detected in peripheral blood of patients with PD, further evidencing an increased inflammatory profile in these patients (Qin et al., 2016). Dopamine (DA) has been demonstrated to influence immune cell function in the brain and here we explore the role of B cells in PD pathogenesis as well as the influence dopaminergic loss and replacement therapies have on B cell populations.

Blood–brain barrier disruption has been confirmed in patients with PD, allowing entry of otherwise non-CNS privileged cells (Kortekaas et al., 2005), including adaptive immune cells. B cell presence has yet to be determined in the PD brain (Brochard et al., 2009; Schonhoff et al., 2020). Still, studies have detected α-synuclein antibodies in the brain (Orr et al., 2005) and aggregation around dopaminergic neurons (Orr et al., 2005). This suggests peripheral B cell involvement via antibody production, improving efficiency of α-synuclein removal via Fc gamma receptor (FcγR) mediated phagocytosis. As a result, their role in disease is gaining interest among the scientific community, even spurring the development of immunotherapies. These include novel monoclonal antibodies against the C terminal of α-synuclein such as 1H7 or vaccination with short peptides (AFF1), causing anti-α-synuclein antibody production (Bae et al., 2012; Games et al., 2014; Mandler et al., 2015; Spencer et al., 2017). Some therapies have even reached human trials, including PRX002/RG7935 (PRX002), a humanized monoclonal antibody targeting aggregated α-synuclein to prevent neuronal transfer of pathogenic aggregates (Jankovic et al., 2018). In patients with PD, there may be single nucleotide polymorphisms (SNPs) in immune related genes, conferring an increased inflammatory phenotype. The rs3129882 SNP has been shown to be associated with PD (Kannarkat et al., 2015) and is found within the MHC-II gene locus. This SNP upregulates MHC-II expression in B cells and monocytes, which increases their capacity for antigen presentation (Wang et al., 2022). Other genes associated with MHC-II, including HLA-DRB5 (Arlehamn et al., 2017), HLA-DQA2 (Wissemann et al., 2013), and HLA-DPB1 (Bi et al., 2021) were also found to be upregulated in memory B cells of patients with PD, making it likely that B cells enhance inflammation (Schonhoff et al., 2020) by increased antigen presentation to T cells.

Interestingly, in the blood sera of patients with PD, antibodies reactive against α-synuclein and its derivatives were elevated in those with early disease onset (5 years), identified as being fourfold higher than controls (Gruden et al., 2011). There was also an increase in autoantibodies against DA, which were 25-fold higher than in age matched controls. Both of these levels decreased in late disease (10 years), as detected by ELISA, consistent with other studies (Besong-Agbo et al., 2013), however the cause remains undiscovered. B lymphocytes were also reduced in patients with PD compared to controls, detected via flow cytofluorometric analysis, indicating a possible link between disease and adaptive immunity (Gruden et al., 2011). This decrease in anti-α-synuclein antibodies and DA antibodies with disease progression could be attributed to reduced B cell activation by Th2 CD4 cells (Kustrimovic et al., 2018). Another possibility could be due to an increase in oxidative stress as the disease progresses, as a result of inflammatory processes directly associated with α-synuclein accumulation (Giasson et al., 2000). At the early stages of disease, these autoantibodies may protect dopaminergic neurons from destruction. However, as PD advances, reduced antibody binding to DA results in a relative deficiency of antioxidant activity. Consequently, the immune response is unable to counter oxidative stress and cytotoxicity effectively (Gruden et al., 2011). Free DA then disrupts mitochondrial respiration leading to the release of cytochrome c, and triggering neuronal apoptosis (Berman and Hastings, 1999). The significance of auto-anti-α-synuclein affinity has also been emphasized and reveals lower levels of these high affinity autoantibodies in patients with PD compared with healthy controls. This may result in inadequate clearance of α-synuclein and reduced localization of auto-anti-α-synuclein antibodies, which provides rationale for the lack of α-synuclein accumulation in the healthy brain (Brudek et al., 2017).

B cell numbers are known to be altered in patients with PD, with many studies presenting a decrease in these populations (Kedmi et al., 2011; Kobo et al., 2016), but there are inconsistencies in the literature (Jiang et al., 2017). Others suggest alterations to different B cell populations. *Ex vivo* cytometric analysis of peripheral blood mononuclear cells (PBMCs) from patients with PD determined decreases in B cell proliferation compared to controls (Li et al., 2022). The ratio of Bregs, including transitional B cells were reduced, consistent with other studies (Alvarez-Luquin et al., 2019), which may contribute to disease by reducing suppression of antibody production. In contrast, B cells producing proinflammatory cytokines were increased. Unsupervised principal component analysis also revealed increased TNF-α and GM-CSF expression in both B and T cell populations. Follicular T cells, known to support B cell helper functions, were also reduced (Li et al., 2022). Single cell sequencing in a small patient cohort also detected decreased naïve and increased memory B cells, with clonal expansion of MHC-II alongside transcription activator protein-1, which regulates antigen presentation to T cells (Wang et al., 2022).

A recent study has comprehensively analyzed B cells in both mouse models and patients with PD. B cell numbers were reduced in PBMCs of α-synuclein transgenic mouse models of PD (Thy1 SNCA mice and MI-2 mice), via flow cytometric analysis (Scott et al., 2023). This was confirmed in patients with early PD vs. controls (*n* = 41, each group) especially in those at risk of developing early dementia, determined by microtubule-associated protein tau (MAPT) genotype and neuropsychological predictors (Williams-Gray et al., 2009). B cell depletion effects were also assessed, using a toxin-induced mouse model of PD, involving 6-OHDA injection into the striatum of mice either deficient in B cells (μMT mice) or those that received anti-CD20 antibodies. At 4 weeks post-injection, μMT mice had significantly impacted motor outcomes and greater dopamine loss. Mice that received anti-CD20 antibody also had greater dopamine loss (Scott et al., 2023). These more severe pathological and behavioral outcomes seen with deficient B cells provides evidence that B cells may have an early protective role in mediating dopaminergic cell loss. Furthermore, immunophenotyping of B cell populations in patients with PD determined a reduction in specific lymphocyte populations, including Bregs, defined as CD5+ and CD1d+ cells, observed especially in those with a greater risk of developing early dementia. Reduced Bregs were also associated with increased MDS-UPDRS-III scores indicating worse motor scores. Therefore, increased Bregs seem to be protective in maintaining motor activities and may have a protective role in PD pathogenesis (Scott, 2022). Lastly B cells were stimulated *in vitro* to assess the production of IL-6 and IL-10, which determined no difference in this balance compared to controls, however total cytokine production was increased (Scott et al., 2023). B cells in patients with PD may therefore be more amenable to stimulation and in turn more able to influence the cytokine environment to modulate other cells.

Decreases in B cell populations has also been noted as a result of levodopa therapy. A recent study examining venous blood from 88 patients with PD used flow cytometry and determined a 15% reduction in total CD19+ B cells within 3 months of treatment initiation (Stevens et al., 2012). It is widely recognized that B cells express DA receptors, stimulation of which can inhibit B cell proliferation (Meredith et al., 2006). Peripheral B cell depletion also correlated to disease progression, however, larger patient studies are required to confirm this. If found to be causative, this could be attributed to reduced production and effect of antibodies, leading to increased neuronal cell death and DA release, further inhibiting B cell proliferation. It is also possible that, if B cells are determined to have a proinflammatory effect in the brain, the effect of DA on their receptors, via release from neuronal cells or by DA therapy, could have the added benefit of replacing DA while reducing neuroinflammation.

### 2.4 Alzheimer’s disease

Alzheimer’s disease is characterized by the accumulation of amyloid-β (Aβ) and Tau proteins within the brain, leading to neurodegeneration and cognitive decline (Ittner and Götz, 2011; Ovsepian et al., 2019). Aβ plaques also result in BBB disruption, causing cerebral amyloid angiopathy, which disrupts leptomeningeal walls and the cerebrovasculature, leading to peripheral immune cell infiltration (Chen and Holtzman, 2022). Recently the role of B cells in disease pathogenesis has been explored with conflicting results – some studies suggest a beneficial role (Feng et al., 2023) with IL-35 secreting B cells reducing Aβ deposition and cognitive dysfunction, while others associate B cells with neurocognitive deficits (Kim et al., 2021). B cells have been found to accumulate in the brain parenchyma, resulting in antibody deposits around Aβ plaques (Kim et al., 2021). In the 3xTgAD mouse model, innate B1a B cells show increased frequency in circulation and secondary lymphoid organs, and a higher percentage of cell activation, expressing cytokines like IFN-γ, IL-6, IL-10, and TGF-β. Interestingly these cells show a combination of pro- and anti-inflammatory cytokines similar to the phenotype described in the context of injury (Sîrbulescu et al., 2021; Dwyer et al., 2023). B cells have been described in single cell RNA-seq immunophenotyping in the brains of 5xFAD mice (Keren-Shaul et al., 2017) and infiltrating B cells have been detected in the parenchyma of the frontal cortex and hippocampus. Furthermore, IgG foci have been shown to colocalize with microglia and Aβ plaques. It has been positively shown that B cells contribute to an overactivated phenotype of the endogenous microglia, associated with impaired clearance of Ab and worse neuroprotective outcomes (Kim et al., 2021). Though there is little known about these B cells, if certain subsets that contribute to this pathogenicity are identified, a new approach could involve targeted depletion therapy, similar to disease modifying therapies for MS.

Some studies indicate a neuroprotective role for B cells, likely mediated by the production of anti-Aβ antibodies which aid in the removal of Aβ plaques. Aβ vaccination in APP + PS1 mice has been shown to improve performance in working memory tests compared with control transgenic mice (Morgan et al., 2000). In Rag-5xFAD mice, deficient in T, B, and NK cells, Rag deficiency results in reduced non-specific immunoglobulin and reduced clearance of Aβ, compared to immunocompetent 5xFAD mice. Rag-5xFAD mice adaptive immune cell reconstitution via bone marrow transplant showed reduced AD pathology, suggesting a role of the peripheral immune system in disease pathogenesis (Marsh et al., 2016). In plasma and CSF samples from patients with AD and healthy controls, IgG targeting oligomeric Aβ1-42 decreased with both age and AD progression, suggesting increasing immune dysfunction in these populations. Furthermore, naturally occurring IgGs extracted from the plasma of patients with AD and healthy controls were shown to reduce Aβ-induced primary neuron toxicity in hippocampal neuronal cultures treated with Aβ oligomers vs. control (Britschgi et al., 2009). Therefore, these antibodies have been suggested to have therapeutic potential, encouraging microglial phagocytic activity against Aβ *in vitro* (Gold et al., 2013). This also reflects the recent approval of the Aβ-targeting drug aducanumab by the FDA, however clinical results are still controversial (Alexander et al., 2021; Rabinovici, 2021; Song et al., 2022). Tau-targeting antibodies are also an attractive option in AD treatments, but there is a lack of consensus whether these antibodies are increased in patients with AD, with statistically significant differences in mild cognitive impairment only detected between genders in AD (Krestova et al., 2018). Additionally, clinical trials using IVIG from healthy controls have not yielded beneficial outcomes, resulting in termination of Phase III clinical trials in patients with AD (Relkin, 2014; Relkin et al., 2017; Okuya et al., 2018).

### 2.5 Amyotrophic lateral sclerosis

Amyotrophic lateral sclerosis is a neurodegenerative condition with a prevalence of approximately 6 cases per 100,000, characterized by progressive degeneration of motor neurons in the brain and spinal cord. This condition has an average age of onset of 58–60 years and the average survival from onset to death is 3–4 years (Riva et al., 2016; Talbott et al., 2016). The etiology of ALS is not well understood, and likely multifactorial. Although over 50 disease-modifying genes have been identified in familial ALS, including variants in SOD1, C9ORF72, FUS, and TARDBP (Boylan, 2015), approximately 90%–95% of ALS cases occur sporadically (Chen et al., 2013). It has been proposed that immunological changes including a reduction of the T regulatory compartment may be associated with a more aggressive disease (McCombe and Henderson, 2011; Malaspina et al., 2015; Beers et al., 2017; McCauley and Baloh, 2019). Proteomic analysis of plasma and PBMCs in blood samples from patients with ALS has shown the activation of molecular pathways involved in immunoregulation and cell senescence in faster progressing ALS (A–F) and at a later stage of disease (Zubiri et al., 2018).

In clinical samples from patients with ALS, high parameter immunophenotyping of whole blood has identified distinct immune profiles potentially associated with different disease subtypes. Interestingly, a relative increase in the lymphocyte compartment, including B cells and T cells was associated with a survival benefit of 160 weeks (87%) as compared to immune profiles that were more similar to healthy controls (Gustafson et al., 2017).

The role of B cells in the context of ALS is not well explored and has only been investigated tangentially in immunophenotyping studies. A preclinical study in the SOD1*^G93A^* mouse model of ALS indicated that neither the phenotype nor physiology of B cells is altered by this mutation (Naor et al., 2009). However, the results of the latter study suggest a detrimental impact of B cell depletion on overall survival (Naor et al., 2009). Interestingly, Pennati et al. (2016) demonstrated that intravenous delivery of IL-10+ Bregs decreased myeloid-derived macrophages in the CNS of SOD1*^G93A^* mice and did not significantly prolong survival, only modestly improving rotarod performance compared to the delivery of control splenocytes once a week for 3 weeks every month. In addition, a case report of a single patient who received Rituximab for treatment of ALS demonstrated no reported benefit (Li et al., 2023).

Study of the role of B cells in neuroinflammation and immune dysregulation in ALS is clearly warranted, both to explore novel biomarkers of progression and to define new immune therapeutic targets. Furthermore, the emerging complexity of the immune dysregulation observed in ALS that involves both adaptive and innate immune populations would support the view that therapeutically targeting the immune system in this disease, and in particular B or T cell function in that context, will be challenging. The rigorous definition of immune dysregulation, which would include dysregulated interactions between innate and adaptive immune cell subpopulations in ALS is clearly needed in both longitudinal high dimensional immunological studies of patients and animal models of the disease. First steps are being made in this regard including a recent study of patients at the time of ALS diagnosis which revealing specific T cell subpopulations in the blood and CSF, including Tregs, which were associated both phenotypically and functionally with the rate of disease progression (Yazdani et al., 2022). Similarly, Yildiz et al. (2023) demonstrated an elevation of senescent and late memory T and B lymphocytes in the blood as a feature of faster progressing ALS and of ALS individuals with bulbar involvement. Clearly the definition of causation beyond simple association in the context of immune dysregulation and the role of B cells in ALS remains an elusive goal at this time.

## 3 B cells in acute injuries of the CNS

The roles of B cells in the context of acute injury of the CNS have been recently reviewed elsewhere (Maheshwari et al., 2023). Here, we will focus on some of the key pathways emerging from recent studies that used experimental manipulations of B cell numbers to modulate inflammation after acute injury such as traumatic brain injury (TBI), spinal cord injury (SCI), and ischemic and hemorrhagic stroke.

### 3.1 Traumatic brain injury

Current understanding suggests that B cells aid in the recovery process after TBI by releasing anti-inflammatory cytokines like TGF-β and IL-10. In an acute stab wound brain injury model, both ligands and target genes for TGF-β showed increased activity in the injured tissue, and led to reduced activation in reactive astrocytes and microglia (Divolis et al., 2019). This mechanism helps limit the overactivity of astrocytes and microglia. B cell deficient μMT^–/–^ mice exhibit a more intense immune response after a TBI, with a marked increase in CD11b+ monocytes and DCs at 8 weeks (Daglas et al., 2019). This suggests that B cells might offer protection against such heightened inflammatory reactions. In the brains of B cell deficient mice, there was also an increase in the numbers of CD4+ T cells and CD69+ activated NKT cells, suggesting that the presence of B cells may modulate the inflammatory T cell response under normal conditions (Daglas et al., 2019). Notably, in comparison to wild-type mice, the B cell deficient group showed neurological and functional impairments, like tail weakness, an irregular walk, and spasms, up to 12 weeks earlier. Interestingly, in patients with TBI there was a significantly higher frequency of activated B cells in peripheral blood at 7 days after brain injury as compared to healthy volunteers, as well as significantly fewer IL-10+ B cells on day 1 and day 7 after injury (Chenouard et al., 2015).

Direct exogenous B cell therapy has been explored in controlled cortical impact (CCI) models of TBI via intraparenchymal delivery (Sîrbulescu et al., 2019, 2021; Dwyer et al., 2023). Following a CCI, a single direct injection of mature naïve B cells significantly improved cognitive functional recovery in a variety of neurobehavioral paradigms (Sîrbulescu et al., 2019). B cell therapy in this context significantly reduced functional impairments post-injury, including learning and memory as well as motor deficits (Sîrbulescu et al., 2019). Histological assessments found that intraparenchymal delivery of exogenous B cells was associated with a decrease in lesion volume by 40%–60% and with significant reduction in reactive astrogliosis and microglial activation 35 days post-CCI (Sîrbulescu et al., 2019). The mechanisms of B cell response in this context are mediated, at least in part, by MyD88-dependent TLR signaling through TLR2/6 and TLR4 (Sîrbulescu et al., 2021). Flow cytometry analysis of brain biopsies at various time intervals after injury and treatment showed that 20%–30% of the exogenous B cells respond to the injured microenvironment by producing a complex cytokine signature that includes classical regulatory cytokines such as IL-10, IL-35, and TGF-β, as well as inflammatory cytokines including TNF-α, IL-6, and IFN-γ (Sîrbulescu et al., 2021; Dwyer et al., 2023). This response pattern of B cells to an injured microenvironment has been termed pligodraxis, reflecting the complex and sequential dynamics of activation and immunomodulatory cytokine production (Sîrbulescu et al., 2021). In TBI, within 24–48 h after transplantation, exogenous B cells showed increased expression of IL-10, IL-35, and TGF-β, but also IL-2, IL-6, and TNF-α. Interestingly, after the initial increase, inflammatory cytokines returned to baseline levels in B cells, however regulatory factors such as IL-10 were persistently expressed by a large proportion of the cells (Sîrbulescu et al., 2021; Dwyer et al., 2023). After 10 days *in situ*, B cell subpopulations characterized by expression of IL-10 or TGF-β dominated (Dwyer et al., 2023). Indeed, cytokines such as IL-10 have been confirmed as essential B cell-derived effector molecules mediating neuroprotection, since loss of IL-10 significantly reduced the neuroprotective effect of B cells after TBI (Sîrbulescu et al., 2021).

The presence of exogenous B cells in the injured brain after TBI did not appear to affect the infiltration dynamics of other populations of immune cells into the injured site, but markedly modulated their cytokine response to injury. In B cell-treated animals, significantly more infiltrating myeloid cells, particularly Ly6C+ monocytes/macrophages, produced regulatory cytokines such as IL-10, TGF-β, and IL-35, and fewer produced TNF-α, IFN-γ, and IL-6 as compared to controls, up to 2 months post-TBI. B cell treatment also significantly increased the proportion of infiltrating monocytes/macrophages that expressed markers of alternative activation, such as CD206, and reduced the relative proportion of activated microglia starting at 4 days and up to 2 months post-injury (Dwyer et al., 2023). Interestingly, the presence of peripheral infiltrating monocytes/macrophages was required for the activation and secretion of regulatory cytokines in the exogenous B cells, suggesting a positive feedback mechanism in which regulatory responses are amplified between B cells and myeloid cells in TBI (Dwyer et al., 2023).

### 3.2 Spinal cord injury

Following damage to the BSCB in SCI, peripheral leukocyte infiltration occurs and immune cells can interact with the lesion microenvironment (Zhen-Gang et al., 2022). It is hypothesized that peripheral lymphocytes migrate from both the spleen and lymph nodes, since a decrease in B cell count within these peripheral lymphoid tissues was observed during SCI (Popovich et al., 2001). In contrast to TBI, several studies had identified a detrimental role of B cells after SCI. In an experimental model of spinal cord compression in mice, antibody-mediated depletion of B cells was associated with slowed hindlimb motor dysfunction 1- and 6-h post SCI, and decreased levels of inflammatory molecules including NF-κB, IL-1β, and TNF-α, at the injury site (Casili et al., 2016). Histological analysis showed that B cell depletion also reduced CD4+ and CD8+ T cell infiltration, and astrocyte activation (Casili et al., 2016). In other SCI B cell depletion models, it is hypothesized that antibody-secreting B cells that accumulate in the CSF and at the site of injury impair recovery (Ankeny et al., 2009).

The contrast between the beneficial effects of B cells in TBI and their detrimental impact in SCI may point to a “site specificity” of immune responses based on the location of injury. The acute inflammatory response to a SCI is significantly greater than in the brain, as indicated by the immune activation post-injury (Schnell et al., 1999). The numbers of neutrophils and macrophages at the site of injury were significantly higher in the spinal cord as a consequence of greater BSCB breakdown. As discussed above, the BSCB exhibits greater permeability due to the differential expression of tight junctions and a lower number of pericytes (Ge and Pachter, 2006; Winkler et al., 2012).

Therapeutic administration of B cells has not yet been performed in conjunction with SCI, likely due to the negative association of endogenous B cell populations with injury severity. It will be interesting to determine in future studies whether the administration of exogenous B cells may induce different immunomodulatory programs in these cells, potentially leading to improved outcomes.

### 3.3 Stroke

In both hemorrhagic and ischemic stroke, localized lack of oxygen and nutrients leads to excitotoxicity, rapid necrosis and cell death (Zera and Buckwalter, 2020). Acute cerebral ischemia from stroke damages the capillary wall of the BBB, increasing vascular permeability and the movement of blood into the brain parenchyma (Malone et al., 2022). Around 24–48 h past the ischemic phase, the effects of peripheral tissue damage lead to proteolytic degradation of the endothelial tight junctions and basement membranes of the BBB. Disruption of the BBB allows for peripheral immune cell infiltration into the brain to augment the neuroinflammatory response (Malone et al., 2022). At the same time, tissue necrosis releases DAMPs and cytokines that drain throughout lymphatic vessels to cause an immune response in secondary lymphoid organs.

Infiltrating B cells access the brain through damaged capillaries and across the BCSFB of the ChP (Malone et al., 2022). Once at the site of injury, B cells produce pro- and anti-inflammatory cytokines to exert an influence on the recovery process. A number of studies have explored the impact of B cells on recovery outcomes post-ischemic stroke. After a middle cerebral artery occlusion (MCAO), B-cell-deficient μMT^–/–^ mice exhibited greater infarct volumes, mortality, and more severe functional deficits when compared to WT mice (Ren et al., 2011). Worse outcomes in B cell-deficient mice were associated with immune-driven neuroinflammation, increased number of activated T cells, macrophages, microglial cells, and neutrophils in the MCAO hemisphere. By contrast, a study in B cell-deficient JHD^–/–^ mice found that the MCAO stroke outcomes were not statistically significant compared to WT mice (Schuhmann et al., 2017). These variations in outcomes warrant further examination, and could be due to the different animal models or the extent of the stroke damage.

As in TBI, transplant or adoptive transfer of B cells has been consistently associated with improved outcome in ischemic stroke models (Maheshwari et al., 2023). Exogenous administration of B cells 24 h before or 4 h after MCAO significantly reduced infarct volume in the cortical, striatal, and total hemisphere regions (Bodhankar et al., 2014). This study also highlights the complex interactions of exogenous B cells *in vivo*, and their immunoregulatory roles. When B cells were administered 24 h prior to MCAO a notable rise in regulatory subpopulations was observed, as well as a decline in inflammatory cell numbers, and diminished T cell infiltration (Bodhankar et al., 2014). B cell treatment was associated with an increase in Foxp3+ CD4+ Tregs and Bregs in B cell treatment groups (Bodhankar et al., 2014), as well as a decline in TNF-α in CD11b+ monocytes, while CD3+ and CD4+ T cells exhibited decreased levels of IFN-γ and IL-17, respectively.

Studies in models of stroke also support the importance of IL-10 as a mediator of Breg activity. After MCAO, animals treated with IL-10 deficient B cells exhibited larger infarct volumes (Ortega et al., 2020), while the administration of enriched IL-10-positive B cells resulted in a reduction in ischemic infarct volume and stroke-induced splenic atrophy (Bodhankar et al., 2014). Interestingly, studies in stroke also found that B cells can actively promote neurogenesis, enhance cell viability, and promote dendritic branching. Using DCX+ and BrdU cells as indicators of neurogenesis, an increased number of DCX+ neuroblasts were observed in the olfactory bulbs and dentate gyrus of WT animals compared to B cell-depleted mice (Ortega et al., 2020). B cells are the primary lymphocytic sources of BDNF, and they appear to modulate this critical factor to support neuronal function (Vega et al., 2003). It is hypothesized that in stroke, the neurotrophic capacity of infiltrating B cells at the post-stroke injury site induces early protection from ischemic injury.

## 4 Aging and the role of B cells in neuroinflammation

Early *in vitro* studies of bone marrow cultures from young and aged mice have shown a notable decrease in the cell surface expression of B220, a B-cell lineage-specific marker, in aged bone marrow (Zharhary, 1988). This reduction was attributed to a deficiency in supportive cell factors within the aged bone marrow, rather than suppressive activities from other immune cells. Hematopoietic stem cells of older mice display a shift toward myeloid-biased clones, resulting in decreased lymphoid populations. Concurrently, diminished IL-7 response in pro-B cells and reduced B cell immune efficacy were observed (Stephan et al., 1997, 1998). The observed reduction in B cell numbers may also be associated with the decrease in B cell precursors caused by reduced surrogate L chains in senescent B cell precursors and compromised pre-BCR checkpoints (Alter-Wolf et al., 2009a,b). Notably, age-associated changes in immune populations have been correlated with autoimmune diseases, especially through the emergence of atypical B cells, termed ABCs (Haas, 2023). ABCs are characterized by T-bet and CD11c expression and have been shown to exhibit selective amplified responses to stimulation via TLR7/TLR9 but not TLR2/TLR4 (Hao et al., 2011; Rubtsov et al., 2011). Moreover, upon TLR stimulation *in vitro*, ABCs demonstrate a preferred release of IL-4 and IL-10, suggesting a distinct inflammatory response pathway. A hallmark feature of ABCs is their increased antigen-presenting capacity, likely stemming from elevated MHC-II levels (Rubtsov et al., 2011). The direct impact of ABCs in the CNS remains understudied, and comprehensive *in vivo* studies are essential to elucidate the broader physiological implications of ABCs in neurodegenerative disorders associated with aging.

## 5 Conclusion

As the understanding of B cell’s multifaceted roles in the CNS deepens, several pivotal directions emerge for future research. Firstly, the exact mechanisms through which B cells modulate neuroinflammation and interact with other immune cells needs elucidation. This includes discerning the balance between their neuroprotective and potentially pathogenic activities. Secondly, the specific signaling pathways and molecular triggers governing B cell function within the CNS present fertile ground for investigation. Tailoring B cell therapies through genetic or pharmacological means may enable more precise interventions. Lastly, understanding the context-specific effects of B cells across various CNS pathologies will be instrumental. As we harness this knowledge, the potential of B cells as therapeutic agents in the CNS will likely transition from experimental to standard care, revolutionizing how we address neurological conditions and injuries.

## Author contributions

JA: Conceptualization, Investigation, Writing – original draft, Writing – review & editing. MM: Conceptualization, Investigation, Writing – original draft, Writing – review & editing. RK: Writing – original draft, Writing – review & editing. YX: Visualization, Writing – original draft. MP: Funding acquisition, Writing – original draft, Writing – review & editing. RS: Conceptualization, Funding acquisition, Investigation, Project administration, Supervision, Visualization, Writing – original draft, Writing – review & editing.
